# A view from the clinic – Perspectives from Dutch patients and professionals on high myopia care

**DOI:** 10.1111/opo.13091

**Published:** 2023-01-17

**Authors:** Monica Ravenstijn, Gerlof du Bois, Ritsert C. Jansen, Chang Liu, Gregorius P. M. Luyten, Redmer van Leeuwen, Mor M. Dickman, Nic J. Reus, Suzanne Yzer, Caroline C. W. Klaver

**Affiliations:** 1 Rotterdam Ophthalmic Institute, the Rotterdam Eye Hospital, Rotterdam, the Netherlands; 2 Department of Ophthalmology, Erasmus University Medical Center, Rotterdam, the Netherlands; 3 Oogvereniging, Utrecht, the Netherlands; 4 Groningen Bioinformatics Centre, University of Groningen, Groningen, the Netherlands; 5 Department of Ophthalmology, Leiden University Medical Center, Leiden, the Netherlands; 6 Department of Ophthalmology, University Medical Center Utrecht, Utrecht, the Netherlands; 7 University Eye Clinic Maastricht, Maastricht University Medical Center, Maastricht, the Netherlands; 8 MERLN Institute for Technology-Inspired Regenerative Medicine, Maastricht University, Maastricht, the Netherlands; 9 Department of Ophthalmology, Amphia Hospital, Breda, the Netherlands; 10 Department of Ophthalmology, Radboud University Medical Centre, Nijmegen, the Netherlands; 11 Department of Epidemiology, Erasmus University Medical Center, Rotterdam, the Netherlands; 12 Institute of Molecular and Clinical Ophthalmology, University of Basel, Basel, Switzerland

**Keywords:** healthcare, myopia, myopia complications, patients' perspective, professionals' perspective, survey

## Abstract

**Purpose:**

To understand and compare perspectives of patients and professionals on current ophthalmologic care for high myopia, and to identify challenges and future opportunities.

**Methods:**

Self-reported data were collected through two online questionnaires. Patient perspective was obtained from highly myopic members of a patient organisation based in the Netherlands using a 17-item questionnaire consisting of open and multiple-choice questions regarding personal experience with myopia care. The ophthalmologist perspective was obtained from practising Dutch ophthalmologists with a 12-item questionnaire of multiple-choice questions on work-related demographics, myopia care in daily practice and need for improvement. The response rate for patients was 27% (*n* = 136/500) and for ophthalmologists, 24% (*n* = 169/716).

**Results:**

Patients were highly concerned about personal progressive loss of vision (69%) and feared their psychological well-being (82%) in case this would happen. The quality of performance of care provided by ophthalmologists was rated as excellent or satisfactory by 64% of the patients. These ratings for multidisciplinary care and insurance reimbursement were as low as 28% and 18% respectively. The mean concern among ophthalmologists about the rise in high myopia was 6.9 (SEM 0.1) on a 10-point scale. Sixty-nine per cent of the ophthalmologists reported that asymptomatic myopic patients should not be examined regularly at outpatient clinics. Ophthalmologists urged the development of clinical guidelines (74%), but did report (95%) that they informed patients about risk factors and complications. This contrasted with the view of patients, of whom 42% were discontent with information provided by ophthalmologists.

**Conclusions:**

These questionnaires demonstrated that the current clinical care delivered to highly myopic patients is in need of improvement. The expected higher demand for myopia care in the near future requires preferred practice patterns, professionals specifically trained to manage myopic pathology, accurate and comprehensive information exchange and collaboration of in- and out-of-hospital professionals across the full eye care chain.

**Supplementary Information:**

The online version of this article (doi:10.1111/opo.13091) contains supplementary material, which is available to authorized users.

## Key points

- Guidelines for myopia management are needed to improve quality and appropriateness of care, to improve cost-effectiveness of interventions, to serve as educational guidance and to identify pertinent research directions.
- Discrepancy between patients and professionals on their judgement of the quality of information exchange can be resolved with better availability of information on risks and treatment options.
- Increased cooperation between ophthalmic care professionals across the entire field of ophthalmic care plus the formation of multidisciplinary teams around complex myopic cases will benefit patients.

## INTRODUCTION

The prevalence of myopia has significantly increased and is now endemic in most of the industrial world.^1^ Following Asia, the prevalence in Europe has risen dramatically from 25% of the young adults 30 years ago to 50% of young adults today.^2^ In parallel with this rise, the prevalence of high myopia (refractive error ≤ −6 dioptres) is estimated to augment to 9.8% of the world's population by 2050.^1^ Although all degrees of myopia are at considerable risk of ocular complications, in particular the excessive axial elongation occurring in high myopia increases the risk of myopic macular degeneration, glaucoma, posterior subcapsular cataract and retinal detachment.^3^ Less common retinal pathology associated with high myopia includes macular holes, foveoschisis, multifocal choroiditis and punctate inner choroidopathy.^4–7^ High myopia also faces surgical challenges, such as inaccurate refractive predictability and risk of retinal detachment after cataract extraction.^8–11^ Taken together, patients with high myopia often have multiple ocular pathologies with limited options and success of treatment, and are therefore at serious risk of severe visual impairment or blindness.^12,13^ Needless to say, these complications can seriously impact the quality of life.^14,15^ How much the fear of this potential burden affects patients' well-being is currently unclear.

On the professional level, the increase in myopia prevalence will undoubtedly affect patient management. In coming years, ophthalmologists will see more myopic complications, often more than one per patient. With the current movement towards subspecialty care, doctors may not be aware of the presence or risk of disorders outside their direct field of expertise. Whether professionals prepare for the upcoming rise in myopic complications and anticipate clinical or organisational problems is unknown.

This study aimed to investigate the patients' and ophthalmologists' perspective on current and future care of high myopia in the Netherlands, with the goal to transform insights into propositions for clinical guidelines.

## METHODS

The study had an exploratory, cross-sectional design and consisted of a self-reported survey among highly myopic patients and clinical ophthalmologists. Personally identifiable information was not collected.

### Patient survey

From July to August 2022, a patient organisation (Oogvereniging, oogvereniging.nl) initiated a survey among all 500 adult members who were either highly myopic patients or parents of young children with progressive (high) myopia from the Netherlands. The survey instruments are provided in the Appendix S1. The survey was structured into 17 items of open and multiple-choice questions available in the Dutch, English, German and French languages. Domains included personal experience, perceptions of current myopia care in adults and children, opinion on need for improvement of care and on priorities for research. The patient survey was distributed using Microsoft Form (Microsoft.com) on Facebook and through newsletters of the patient organisation.

### Ophthalmologist survey

From September to October 2022, we sent out a survey to the 716 Dutch ophthalmologists in active clinical practice; all were members of the Dutch Ophthalmologic Society (Nederlands Oogheelkundig Gezelschap). Data collection took place via SurveyMonkey (surveymonkey.nl). Survey instruments are provided in the Appendix S1. The survey consisted of 12 multiple-choice questions; domains included were work-related characteristics, perceptions of current myopia care and opinion on the need for improvement of eye care. The survey was distributed by e-mail in three waves.

### Statistical analysis

Data from participants were analysed for distributions including mean and standard error of the mean (SEM) where appropriate. With respect to the patient survey, answers to open questions were categorised by six board members of the patient society. Answers to multiple-choice questions were on a 5-point scale ranging from ‘insufficient’ (1) to ‘excellent’ (5). For the ophthalmologist survey, answers to the multiple-choice questions were rated on a 10-point scale, ranging from ‘not at all’ (1) to ‘extremely’ (10). Ratings are provided as means and top-2-box percentages. One-way analysis of variance (ANOVA) was used to compared the mean concerns between ophthalmologists' subgroups. A *p*-value of <0.05 was considered statistically significant.

## RESULTS

### Patient survey: Characteristics and concerns

**FIGURE 1:**
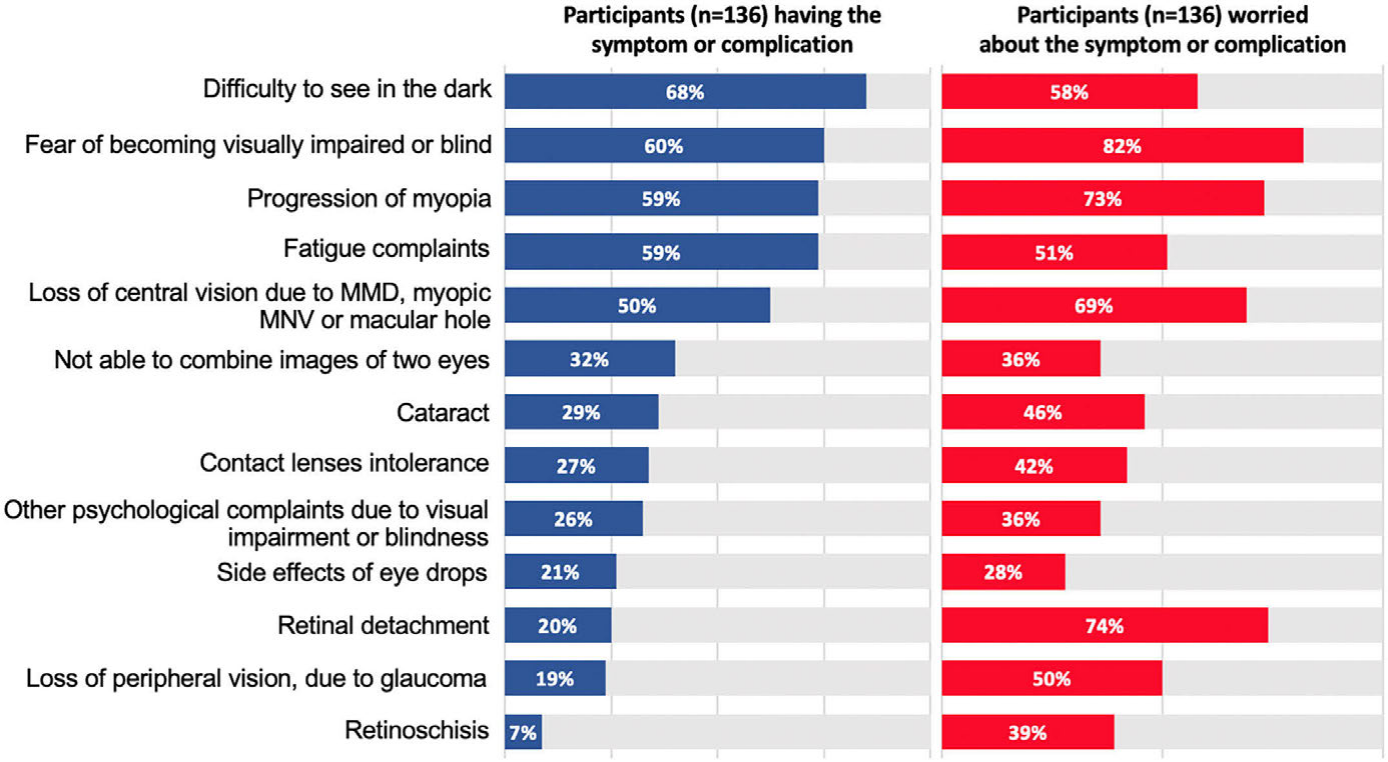
Key respondent characteristics and concerns from patient survey. MMD, Myopic macular degeneration; MNV, Myopic neovascularization.

In this survey, 136 persons from the Netherlands responded to the request for participation. Using standards for response-rate calculation and reporting developed by the American Association for Public Opinion Research,^16^ we calculated a 27.2% response rate. Of the participants, 85% (*n* = 117) were highly myopic adults or adolescents and 15% (*n* = 19) were parents of myopic children. Their visual symptoms or concerns are provided in Figure 1. Most reported were night blindness (68%), fatigue (59%) and progression of myopia (59%). Another issue was intolerance to contact lenses (27%). Vision loss was present in 69% and originated from myopic macular degeneration, neovascularisation, macular hole formation, glaucoma or a combination thereof. Future progressive vision loss was a major concern among an even larger proportion (69%). When asked about the fear of becoming blind, 60% reported current sufferings, while 82% expressed concerns about their psychological well-being when that would happen.

### Patient perspective on current myopia care

**FIGURE 2:**
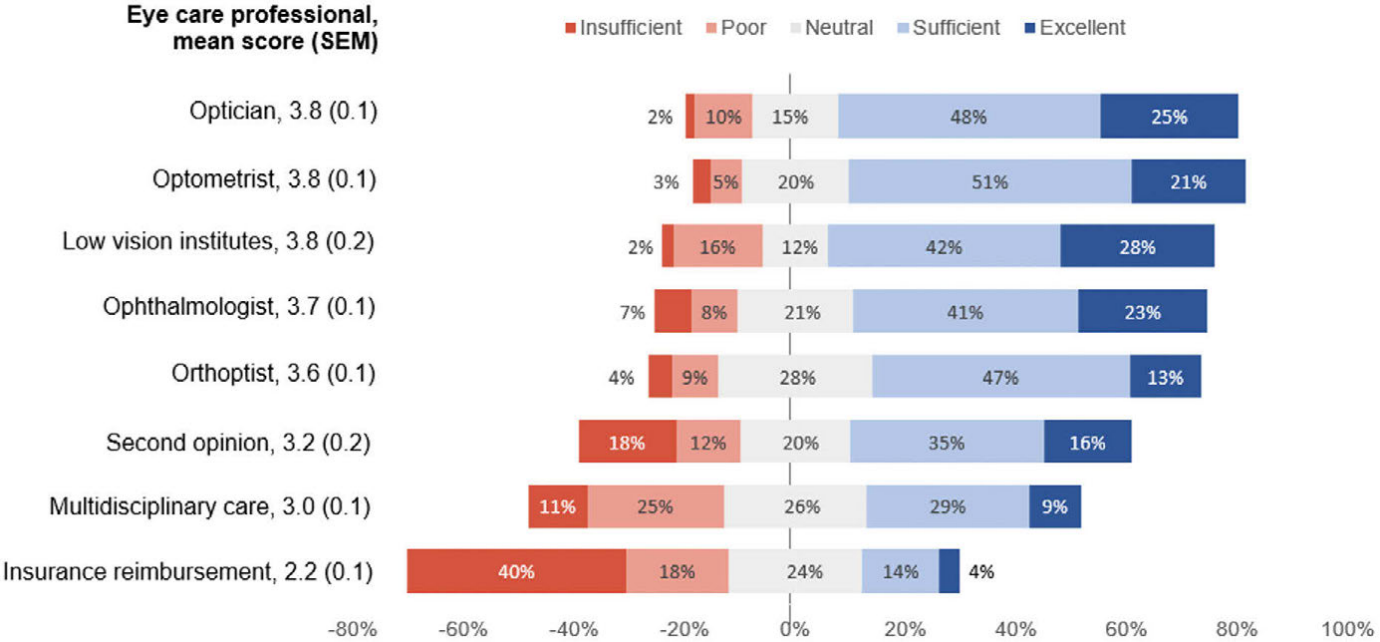
Patient ratings of quality performance of eye care professionals.

Figure 2 provides the scores on quality of care. Mean scores for quality of performance of professionals ranged from 3.2 to 3.8; opticians, optometrists and low vision experts had the highest proportion of ‘excellent’ and ‘satisfactory’ ratings, that is 73%, 72% and 70%, respectively. Ophthalmologists followed with 64%. Quality of multidisciplinary care (MDC) and insurance reimbursement had the lowest proportion of high scores, 28% and 18%, respectively. Forty per cent (*n* = 54) of patients rated the insurance reimbursement as ‘insufficient’.

**FIGURE 3:**
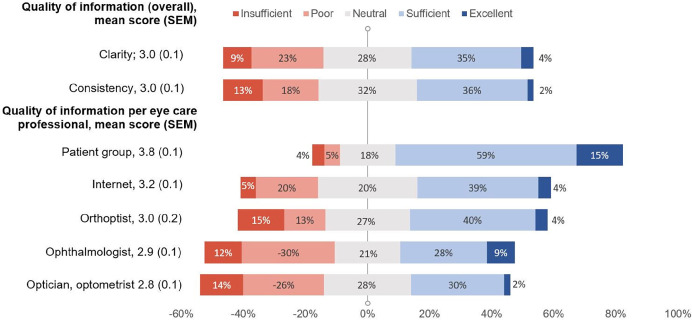
Patient ratings of quality of information exchange through various sources.

Mean scores for information exchange ranged between 2.8 and 3.8; ratings on the quality of various sources are presented in Figure 3. Information offered by the patient group had the largest proportion of ‘excellent’ and ‘sufficient’ ratings (74%). Information provided by ophthalmologists and opticians was scored as ‘poor’ and ‘insufficient’ in a relatively high proportion (42% and 41%, respectively).

### Patient suggestions for improvement

In text boxes for response to open questions, patients shared their positive experiences with eye care (95%, *n* = 129), suggestions for improvements (93%, *n* = 127), desires for MDC (70%, *n* = 95) and requests for insurance reimbursement (60%, *n* = 82). These views included:

- High myopes need clinical consultations regularly, especially complex cases.
- Consultations should allow ample time for information exchange.
- Clarity on risks of myopic complications and treatment options is highly desired.
- Patients request shared decision-making.
- Doctors should be empathic to psychological and social impact of complications.
- Patients need information about low vision care in an early stage.
- Patients urge doctors to organise MDC around complex cases.
- Patients favour development of myopia expert centres.
- Insurance policies should cover costs for treatment and myopia control.

Of the 136 participants, 116 (85%) shared their thoughts on the areas that need development and research:

- control of myopia progression in very young, highly myopic children;
- halt of vision deterioration in highly myopic adults;
- comorbidity of high myopia with other diseases;
- psychological and social impact of high myopia in children and adults;
- patient-friendly aids, tools and treatments;
- (inter)national guidelines for myopia care, which include latest insights;
- decision aids for shared decision-making;
- education of current and next-generation eye care professionals; and
- school systems that include measures for myopia prevention.

### Professional survey: Characteristics and concerns

Of the 716 invited ophthalmologists, 169 (23.6%) responded to the invitation to complete the survey. Work-related demographics of the participants are given in Table 1. Subspecialties were predominantly medical retina (25%), cornea (16%) and glaucoma (14%). Mean concern about the increasing prevalence of (high) myopia was rated as 6.9 (SEM 0.1) out of 10; somewhat higher for vitreoretinal surgeons (mean 7.4, SEM 0.3; *p* 0.55) than for refractive surgeons (mean 5.8 SD 2.6) or uveitis specialists (mean 6.1, SEM 0.5). The concern in more general ophthalmologists who practised within a subspecialty field less than 20% of their worktime was significantly higher than for subspecialists who practised more (20%–90%) within their field of expertise (7.3 vs. 6.3, *p =* 0.02). Differences between subspecialty (*p =* 0.55), type of practice (*p =* 0.56) or years of working experience (*p* = 0.27) were not statistically significant.

**TABLE 1:** Overview of ophthalmologists' work-related demographics.

Variable	*N* (%)	Concern; ratings 1–10	*p* *
Generalist or specialist			0.02*
General ophthalmologist	39 (23)	6.9 (0.3)	
Ophthalmologist with subspecialty (<20% of the time)	41 (24)	7.3 (0.2)	
Ophthalmologist with subspecialty (20–90% of the time)	44 (26)	6.3 (0.3)	
Ophthalmologist with subspecialty (>90% of the time)	44 (26)	7.2 (0.2)	
Subspecialties (*n* = 130)			0.55
Medical retina	28 (22)	7.0 (0.4)	
Cornea and anterior segment	23 (18)	7.1 (0.4)	
Glaucoma	17 (13)	7.1 (0.4)	
Paediatric ophthalmology and strabismus	18 (14)	6.9 (0.2)	
Vitreoretinal surgery	13 (10)	7.4 (0.3)	
Refractive surgeons	6 (4)	5.8 (1.1)	
Others^a^	24 (18)	6.4 (1.9)	
Type of practice			0.56
University hospital	35 (21)	7.1 (0.3)	
General hospital	92 (54)	6.7 (0.3)	
(Private) group practitioners	52 (31)	7.1 (0.2)	
Specialised ophthalmic hospital	8 (5)	7.3 (0.3)	
Work experience			0.27
<5 years	34 (20)	6.5 (0.3)	
6–10 years	35 (21)	6.7 (0.3)	
11–20 years	54 (33)	7.2 (0.2)	
21–30 years	27 (16)	7.3 (0.2)	
More than 30 years	18 (11)	6.8 (0.1)	

*Note*: The mean (standard error of the mean) degree of concern on the rising prevalence of (high) myopia was calculated per subgroup; rated as ‘not at all’ (1), to ‘extremely’ (10) on a 10-point scale.

^a^ Others: oculoplastic and orbital surgeons, uveitis specialists and neuro-ophthalmologists.

* *p*-Value of one-way ANOVA test.

### Professional perspective on current myopia care

**FIGURE 4:**
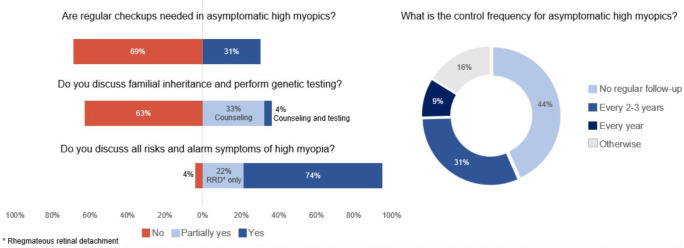
Professional perspective on current myopia care.

The majority of ophthalmologists (69%; *n* = 116) did not feel that highly myopic individuals without any signs of complications should receive a referral to their clinics (Figure 4). When asked about the frequency of consultation, 45% (*n* = 75) answered not to schedule a follow-up visit for these patients, and accepted only consultations for acute visual symptoms. Thirty-two per cent (*n* = 54) of ophthalmologists did check highly myopic patients every 2–3 years, and 9% (*n* = 16) scheduled an annual visit. Some practitioners (*n* = 9) mentioned that the frequency of consultation depended on age, refractive error, ocular symptoms and/or family or medical history. Others (*n* = 5) stated that this depended on the appearance of the optic disc and macula. Sixty-four (37%) ophthalmologists informed patients about the possible inheritance of high myopia, and seven (4%) conducted genetic testing in case of suspicion of a hereditary component.

### Professional suggestions for improvement

Ophthalmologists were asked how to manage the expected increase in myopia and the correlated complications. In descending order, ophthalmologists wished for clinical guidelines or a preferred practice pattern (74%, *n* = 125), easily accessible information via the internet, videos and/or flyers for patients on the risks of myopic complications (70%, *n* = 118), education for eye care professionals (33%, *n* = 56), better reimbursement of myopia care (24%, *n* = 40), more time per consultation (22%, *n* = 37) and dedicated myopia expert centres for complex cases (15%, *n* = 35). Six ophthalmologists (4%) suggested that optometrists and opticians be trained for uncomplicated high myopia screening (*n* = 6). Nine ophthalmologists (5%) emphasised the importance of preventing the development of (high) myopia in children.

## DISCUSSION

Myopia is a rapidly increasing health problem and currently the second largest cause of blindness worldwide.^17^ We investigated views from patients and professionals on myopia care with the aim of developing strategies for the challenges that will be faced in the near future. Both groups underlined the importance of guidelines for myopia management and information on the risk of complications and visual loss. The ratings of patients for current quality of care were markedly lower than professionals had given themselves. In particular, MDC scored low, with almost one-third of patients rating this as ‘poor’ or ‘insufficient’. Patients also indicated that the clarity and consistency of the provided information on high myopia risks and treatment options should improve.

### Myopia concern

In this survey, it is clear that highly myopic patients have a great fear of severe vision loss and blindness. From personal experience, they understand that an increase in the number of cases will have a significant effect on healthcare. By contrast, Dutch ophthalmologists were only moderately concerned about the rising prevalence of myopia and its extreme forms. A mean concern of 6.8 on a 10-point scale in this study was even lower than a previous survey finding the globally lowest score for Australian professionals, with a value of 7.6.^18^ The degree of concern was very similar for subspecialties, type of practice or the years of experience, indicating that this is a generally accepted view of eye care professionals in the Netherlands. It is interesting that the concerns of Asian counterparts are far greater (9.0/10) than those of Europeans.^18^ This is understandable given the much higher rates in Asia, but one may wonder whether eye care in Europe faces enough awareness of the myopia morbidity that has started to expand. Retinal detachment incidence rates in the Netherlands have already increased by 44%.^19^ A comprehensive understanding of the problem among professionals is needed before policymakers will initiate action plans towards healthcare improvement, prevention and research development.

### Myopia care of today

Highly myopic patients were most content with their opticians and optometrists, and less so with other clinicians, that is the ones checking on eye pathology. With respect to care, patients wished for examinations at regular intervals, a keen eye for the multiplex of potential complications and an up-to-date and realistic assessment of risks and treatment options. Most (66%) ophthalmologists, however, reported that regular check-ups for asymptomatic high myopes were not needed, which may explain why some patients feel misunderstood or lack empathy. All ophthalmologists agreed to check patients with symptoms, but often failed to organise the care for more than one complication adequately and efficiently. Subspecialised clinics often demand separate visits to different doctors, or doctors overlook pathology outside their field of expertise.

The opinion of ophthalmologists varied considerably as regards the genetic counseling in high myopia. A minority (37%) discussed familial inheritance with their patients, and few (4%) performed genetic testing. Ignorance of the clinical benefit of genetic testing appears to be the major reason for this. Myopia is a highly complex trait with ~500 identified common genetic susceptibility loci^20,21^ and many rare variants in exomes of genes.^22,23^ High myopia is the most hereditary form and can accompany Mendelian eye disorders such as retinal dystrophy or connective tissue disease.^24,25^ An important consideration when performing genetic testing is that high myopia may precede other eye pathology occurring in these syndromes, or disguise symptoms thereof.^26–28^ Our survey made it clear that clinicians need to be educated on the merit of comprehensive phenotyping and genotyping when a Mendelian inheritance is suspected.

Another discrepancy between patients and professionals was their judgement of the quality of information exchange. Many patients found the information on risks of complications, vision loss and required actions in case of symptoms inadequate, while almost all ophthalmologists stated that they inform patients about alarm symptoms and risks of, for instance, retinal detachment and choroidal neovascularisation. An explanation for this difference in viewpoint may be the acute versus long-term risks. Ophthalmologists find it important to inform patients on direct visual consequences of myopia, while patients have questions about the entire clinical course. They raise questions such as: ‘What is my visual prognosis?’, ‘How quickly will my vision deteriorate?’, ‘Who can help me with my worries and emotional well-being?’ and ‘Are there behavioural restrictions?’ Answers to these questions require in-depth insight into myopia pathogenesis and epidemiology from the professional and ample consultation time to discuss this with the patient. Both of these requirements are not being met in current clinical care.

### Myopia care of tomorrow

Patients and professionals both had views on improvements for the expertise and organisation of care. They strongly recommended the development of clinical practice guidelines for myopia consultations and treatment with the aim of improving the quality and appropriateness of care, to improve cost-effectiveness of interventions, to serve as educational guidance and to identify pertinent research directions. Designing these guidelines may be challenging given the broad clinical spectrum of high myopia and the diverse team of experts that are involved.^3^ Our study showed that current clinical management varied widely, some ophthalmologists scheduled annual visits, while others did not schedule follow-up visits at all. As myopic complications are strongly associated with age and axial length,^29^ it seems prudent to create evidence-based guidelines which relate recommendation for age, axial length, intraocular pressure and medical history. Apart from the frequency of consultation, topics that should be covered in these guidelines include diagnostics and treatment of complications such as myopic neovascularisation and glaucoma, genetic counselling and testing and indications for ocular surgery.

Information exchange by professionals was another field for improvement. As the myopia field is developing rapidly and insights into patient profiles, risks and treatment options undergo continuous advances, keeping doctors up to date is challenging. Interestingly, patients reported that information exchange via the internet was the second most satisfying. Launches of podcasts, video platforms or comprehensive websites may help distribute information as well. Professional and patient organisations could identify the topics that need attention, and ask for information exchange at various levels. An example of such a platform is our myopia website (myopie.nl), which discusses the risks for patients, treatment options for children and long-term prognosis in two languages. It is beyond doubt that regular refresher courses and extra training for ophthalmologists will advance their knowledge to the state-of-the-art as well.

Organisation of care also deserves attention. Ophthalmologists often work solo; teaming up with a diverse group of subspecialists to diagnose and treat patients for various complications in one visit is not customary practice. To diminish the number of patient visits and thereby increase patient satisfaction and decrease the work load for clinicians and the clinic itself, an MDC model could be applied.^30,31^ This could even be done digitally, as current technology facilitates online evaluation of multi-model imaging. A multidisciplinary team can also find better solutions for complex problems such as the following: “Should you remove a cataract at 40 years of age?” or “Should we laser peripheral lattice in a patient without a posterior vitreous detachment?” The benefits of the MDC model are that: (1) patients can be treated by their own ophthalmologist with advice from a MDC team; (2) patients can be registered in a MDC database which can be used for research and (3) patients have access to surgeons with ample experience in surgery of the myopic eye.

Last but not least, screening of highly myopic patients for the presence of complications does not have to take place in a hospital setting. Out-of-hospital care facilities where trained opticians and/or optometrists perform examinations and imaging in high myopes at regular intervals could help in dealing with the high load of patients who are still at low risk. Only patients with ‘red flags’, for instance high intraocular pressure or visual loss, would then be referred to an in-hospital ophthalmologist. Tools developed by artificial intelligence could serve as a referral aid. The patients themselves suggested so-called ‘decision aids’, charts that can be shared digitally with the professional that patients can use to prepare themselves for hospital visits and be re-read afterwards. Other trends, such as telemedicine and new technology for self-monitoring (e.g., intraocular pressure measurements at home) can also reduce the pressure on ophthalmic care.^32,33^ The hospitals can then transform themselves in myopia expert centres focusing on complex pathology.

### Insurance reimbursement

This survey revealed that many myopic adults and parents of myopic children are unhappy with the current insurance reimbursement; 82% of the Dutch participants gave a rating of neutral or lower and 40% rated it as insufficient. The costs of measures offering myopia control (e.g., multifocal contact lenses) for children with progressive myopia are often not or only partly reimbursed.^34^ Neither are costs of optimised glasses for adults. Redesigning insurance policies for these necessary measures is very much needed as is reimbursement of costs for longer consultations that allow for thorough examination of complex pathology and sufficient time for information exchange.

### Strengths and limitations

This study has strengths and limitations. A great strength is the combination of patient and professional perspectives, which allows for an intriguing comparison of similarities and differences in views and points out areas for improvement. A limitation is the relatively low response rate which increases the potential for selection bias among participants. Ophthalmologists who feel myopia is an increasing public health issue may have been more eager to complete the survey. Similarly, patients unhappy with their ophthalmologic care or worried about their future could have been more motivated to fill in the patient survey. Although this bias cannot be discarded, the wide range of answers suggests that participants reflect a representative group of myopes and eye care providers.

## CONCLUSIONS

Based upon the findings of the surveys, we feel that the priority for improving myopia care should be on: (1) guidelines for ophthalmologists regarding myopia management; (2) improvement and better availability of information for patients on the risks and treatment options; (3) increased cooperation between ophthalmology care professionals across the entire field of ophthalmic care plus the formation of multidisciplinary teams and (4) more in-depth research into myopia complications and targets for intervention. To provide a clear stimulus for implementing recommendations, several quality indicators for structure, process and outcomes should be defined and monitored, thereby allowing demonstration of an improvement in care. Patients and their advocates are willing to help this exciting endeavour succeed.

## Supplementary Information

Supplementary file (DOCX 21.5 KB)
