# Supplementary material for: A view from the clinic – Perspectives from Dutch patients and professionals on high myopia care
Source: Ophthalmic Physiol Opt. 2023 Jan 17;43(3):327–36. doi: 10.1111/opo.13091 (PMC12852226; doi:10.1111/opo.13091)
Supplement: Supplementary file 1 — Supplementary file (DOCX 21.5 KB) [file 44402_2023_4303006_MOESM1_ESM.docx]

**Appendix A: Patient’s survey**

On behalf of the High Myopia patient group of the Eye Association Netherlands, a survey is being conducted into the experiences in the field of eye care for myopia. Anyone with myopia (nearsightedness), high myopia (strong myopia of -6 or worse) or parents of children who have myopia or are being treated for it are welcome to complete this survey.

The questions are about your experiences with the eye care you have received, what went well and what you think needs to be improved. Healthcare is a broad concept. For example, you can think of the information you receive, the diagnosis, prevention, and treatment, making decisions about this together, the attention for what it does to you, and of course whether you feel well helped.

The results will be presented by the High Myopia patient group itself at the international myopia conference (https://www.internationalmyopiaconference.org) to be held in the Netherlands from September 4-7, 2022. The conference is the worldwide meeting for specialists, researchers & policymakers in myopia management.

The survey provides input for good collaboration between patient and healthcare professional/researcher and thus contributes to improving eye care. A unique opportunity for us as a patient group to ensure that scientists work on the questions that concern us!

The survey is anonymous and will take approximately 7 minutes of your time. Please do not enter any personal information such as name, address, etc. Please complete the survey as soon as possible or no later than Aug 12th.

Complete the questionnaire once. Do you have myopia yourself, and are you also a parent of a child/children with myopia? Then you can fill in the questionnaire twice. Then choose carefully from the start from which position you fill in the questionnaire.

General questions

1. *Required*: I complete this questionnaire as:
   - Parent or caregiver of child(ren) with myopia
   - Adult with myopia

2. I complete this questionnaire based on the care I received in the country:

- Netherlands
- Belgium
- Germany
- France
- Other

Questions about your experiences in eye care

3. How do you rate the care received from the following specialists/therapists? *No experience with a specialist/therapist? Then choose 'N/A'*

|  | Excellent | Good | Neutral | Poor | Bad | N/A |
| --- | --- | --- | --- | --- | --- | --- |
| Optician/contact lens specialist | O | O | O | O | O | O |
| Orthoptist | O | O | O | O | O | O |
| Optometrist | O | O | O | O | O | O |
| Ophthalmologist | O | O | O | O | O | O |
| Low vision institutes (Bartimaeus, Visio, Robert Coppes (NL) or BrailleLiga, Licht en Liefde (Belgium) or a comparable body | O | O | O | O | O | O |
| Referral / second opinion | O | O | O | O | O | O |

4. Do you suffer from or are you worried about myopia in combination with another eye disease or illness? If so, explain below which disease:

_________________________________________________________________

Q5. What is your experience with treatment/advice/guidance from multiple healthcare practitioners? For example, if you have multiple eye conditions and visit multiple specialists or, for example, if you have an eye condition in combination with a non-eye condition: *

|  | Excellent | Good | Neutral | Poor | Bad | N/A |
| --- | --- | --- | --- | --- | --- | --- |
| My experience is : | O | O | O | O | O | O |

Q6. Optional explanation to question 5

__________________________________________________________________

Questions about possible myopia complications and what this does to you. if parent/caretaker answer these about your child(ren).

Q7. What symptoms do you have the most or are you most worried about? *Multiple answers are possible.*

|  | Symptoms, not worried | Symptoms, worried | No symptoms, not worried | No symptoms, worried; | N/A |
| --- | --- | --- | --- | --- | --- |
| Progression of myopia | O | O | O | O | O |
| Tiredness due to decreasing vision | O | O | O | O | O |
| Fear of decreasing vision, blindness | O | O | O | O | O |
| Psychological effects of decreasing vision | O | O | O | O | O |
| No longer able to wear contact lenses | O | O | O | O | O |
| Side effects of eye drops | O | O | O | O | O |
| Difficulty to see in the dark | O | O | O | O | O |
| Cataract | O | O | O | O | O |
| Retinal detachment | O | O | O | O | O |
| Eye floaters and similar conditions | O | O | O | O | O |
| Loss of central vision due to retinal degeneration | O | O | O | O | O |
| Loss of central vision due to bleeding | O | O | O | O | O |
| Loss of central vision due to hole in retina | O | O | O | O | O |
| Loss of peripheral vision due to glaucoma | O | O | O | O | O |
| Not able to combine images of two eyes | O | O | O | O | O |
| Retinoschisis | O | O | O | O | O |

8. How do you rate the reimbursement you receive from your healthcare insurance for the treatment of myopia?

|  | Excellent | Good | Neutral | Poor | Bad | N/A |
| --- | --- | --- | --- | --- | --- | --- |
| Reimbursement: | O | O | O | O | O | O |

9. Additional explanation to question 8

_________________________________________________________________

Questions about what you really like about eye care and what you think could be improved

Questions 11 and 12 are used to talk to researchers about the research agenda of the future. So, your input is much appreciated!

10. What has helped you the most? Describe your three most positive experiences.

___________________________________________________________________

11. What would you like to see improved in eye care? Describe your three recommendations.

___________________________________________________________________

Q12. What areas require (additional) research? Describe your three recommendations.

___________________________________________________________________

Questions on information about myopia

Q13. How do you assess the available information (such as websites, flyers, etc.) and the information about myopia and myopia prevention provided during treatment by the professionals/organisations listed below? No experience with a choice? Then select 'N/A'.

|  | Excellent | Good | Neutral | Poor | Bad | N/A |
| --- | --- | --- | --- | --- | --- | --- |
| Public information on the internet | O | O | O | O | O | O |
| Information provided by Opticians and Optometrists | O | O | O | O | O | O |
| Information provided by Ophthalmology Department Hospital | O | O | O | O | O | O |
| Information provided by Patients' Association | O | O | O | O | O | O |

14. To what extent did the information received through different channels help in making the treatment choice together with the practitioner? *

|  | Excellent | Good | Neutral | Poor | Bad | N/A |
| --- | --- | --- | --- | --- | --- | --- |
| The information helped me | O | O | O | O | O | O |

15. How clear are the advices, and treatment protocols you receive from your healthcare professional or from official information sources? *

|  | Excellent | Good | Neutral | Poor | Bad | N/A |
| --- | --- | --- | --- | --- | --- | --- |
| Clarity is | O | O | O | O | O | O |

16. How consistent are the advices, treatment protocols you receive from your healthcare professional or from official information sources? *

|  | Excellent | Good | Neutral | Poor | Bad | N/A |
| --- | --- | --- | --- | --- | --- | --- |
| Consistency is | O | O | O | O | O | O |

Q17. Optional explanation to question 14, 15 or 16

___________________________________________________________________

Closing

Thank you for participating in this research!

**Appendix B: Ophthalmologist’s survey**

1. How concerned are you of the rising prevalence of (high) myopia on a 10-point scale?
   - 1 not concerned at all
   - 2
   - 3
   - 4
   - 5
   - 6
   - 7
   - 8
   - 9
   - 10: extremely concerned
2. How often do you have high myopic patients (SE < - 6 Diopters) in your practice?
   - Never
   - Up to a few per month (1 to 3 per month)
   - Up to a few per week (3 to 10 per month)
   - Multiple patients per week (> 10 per month)
3. What is your control frequency in highly myopic adults without pathologic changes?
   - No regular follow-up visits, only in case of visual symptoms
   - Control visits every 2 to 3 years
   - Control visits every year
   - Other: _________________________________________
4. Do you think a highly myopic adults, without pathologic changes and a good visual acuity, should visit your practice?
   - Yes
   - No
5. Do you discuss the increased risk of retinal detachment, myopic macular degeneration or glaucoma with your myopic patients?
   - Yes, I discuss all the risks and alarm symptoms (e.g., flashes of light, metamorphopsia, blurred vision)
   - Yes, I discuss the alarm symptoms of a retinal detachment
   - No

Other remarks: ______________________________________

1. Do you discuss the possibility of a genetic inheritance in high myopia?
   - Yes, I tell my patient the possible inheritance.
   - Yes, I tell my patient the possible heritance and conduct genetic testing, if necessary.
   - No
2. Wat is needed, in your opinion, to secure the quality of ophthalmic care in myopic adults? *Multiple answers are possible.*
   - Nothing
   - A preferred practice patterns
   - More education for ophthalmologists
   - More time per consultation
   - Higher insurance reimbursement on myopia care
   - Create expertise centers for complex cases
   - Accessable information for patients on risks, alarm symptoms etc. (e.g., websites, videos, flyers)
   - Other ideas: ­­­­­­­­­­­­______________________________________________
3. Where do you want to learn about?
   - Myopic macular degeneration: diagnostics and follow-up
   - Exudative maculopathy (for instance myopic macular neovascularization): diagnostics, treatment and follow-up
   - Glaucoma: diagnostics and follow-up
   - Myopic traction maculopathy: follow-up and indications for surgery
   - Genetic counseling and testing in high myopia
   - Cataract surgery: who and when to operate?
   - Other ideas: _____________________________________________
   - None of the above
4. In what type of practice do you work?
   - Academic hospital
   - Peripheral or general hospital
   - Private grouped practitioners
   - Specialised ophthalmic hospital
5. How do you describe your work?
   - I’m a general ophthalmologist
   - I’m a general ophthalmologist with a subspecialty less than 20% of the week
   - I’m a general ophthalmologist with a subspecialty 20 to 90% of the week
   - I’m a specialised ophthalmologist more than 90% of the time
6. If you have a subspecialty, which one is your focus?
   - Medical retina
   - Vitreoretinal surgery
   - Oculoplastic and orbital surgery
   - Refractive surgery
   - Cornea and anterior segment
   - Pediatric ophthalmology
   - Glaucoma
   - Neuro-ophthalmology
   - Uveitis
   - Other: ____________________
7. For how many years have you been working as an ophthalmologist?
   - 5 years or less
   - 6 to 10 years
   - 11 to 20 years
   - 20 to 30 years
   - More than 30 years
